# Capsule Commentary on Bremmer et. al., Impact of Procalcitonin Guidance on Management of Adults Hospitalized with Chronic Obstructive Pulmonary Disease Exacerbations

**DOI:** 10.1007/s11606-018-4341-x

**Published:** 2018-02-20

**Authors:** Alexander G. Mathioudakis

**Affiliations:** Division of Infection, Immunity and Respiratory Medicine, The University of Manchester, Manchester, UK

Recent data reveal an alarming 50% increase in mean antimicrobial resistance within the last decade, across the OECD member countries.^1^ Moreover, the results of extensive campaigns against inappropriate antibiotic prescribing are only modest,^2^ emphasizing the need for alternative, effective stewardship methods. Serum procalcitonin, being able to differentiate bacterial versus viral infections or non-specific inflammation, is increasingly and successfully used to guide the initiation and/or discontinuation of antibiotics from primary to intensive care settings.^3^

Safe antibiotic avoidance in COPD exacerbations is of utmost importance: they are prevalent, responsible for > 10% of all hospital admissions^4^; and while < 50% are attributed to bacterial infections, patients frequently receive unneeded antibiotics, while their airways are colonised by bacteria that progressively become less sensitive to antibiotics.^4^ The efficacy and safety of procalcitonin to guide antibiotic administration in COPD exacerbations is supported by a meta-analysis of eight open-labelled clinical trials, which however highlights the methodological limitations and small overall population of the included studies.^3^

Bremmer and colleagues, in a retrospective pre-/post-intervention study assessed the safety and efficacy of the implementation of procalcitonin guidance for antibiotic administration for COPD exacerbations in the real world.^5^ Use of procalcitonin was clearly associated with a decrease in overall antibiotic exposure, without any safety signals. Interestingly, it was also correlated with decreased hospitalisation duration and, consequently, cost savings. That is consistent with previous results^3^ and could be attributed to an earlier discharge of patients when bacterial infection is excluded. However, the study is limited by its small study population (*n* = 305) and, possibly, selection bias, as only patients who had their procalcitonin measured were included in the post-intervention group and clinicians might have omitted procalcitonin in severe presentations, where they would administer antibiotics anyway.

Another important, consistent observation was the relatively limited adherence to procalcitonin guidance, which probably reflects the limited confidence of clinicians on its safety. Indeed, few trials have tested compulsory adherence to procalcitonin guidance for antibiotic administration and none of them assessed COPD exacerbations. Therefore, the conduction of appropriately designed, adequately powered, confirmatory, pragmatic trials is urgently required to allow the introduction of procalcitonin guidance in clinical practice.
